# Differences Between Self-Reported Psychotic Experiences, Clinically Relevant Psychotic Experiences, and Attenuated Psychotic Symptoms in the General Population

**DOI:** 10.3389/fpsyt.2019.00782

**Published:** 2019-10-29

**Authors:** Tais Silveira Moriyama, Jim van Os, Ary Gadelha, Pedro Mario Pan, Giovanni Abrahão Salum, Gisele Gus Manfro, Jair de Jesus Mari, Eurípedes Constantino Miguel, Luis Augusto Rohde, Guilherme Vanoni Polanczyk, Philip McGuire, Rodrigo Affonseca Bressan, Marjan Drukker

**Affiliations:** ^1^Centro de Atendimento Especializado, Instituto Bairral de Psiquiatria, Itapira, Brazil; ^2^Department of Psychiatry, Federal University of São Paulo, São Paulo, Brazil; ^3^Brazilian High-Risk Cohort Study for Psychiatric Disorders, National Institute of Developmental Psychiatry for Children and Adolescents (INCT-CNPq), São Paulo, Brazil; ^4^Department of Psychiatry and Psychology, School of Mental Health and Neuroscience (MHeNS), Maastricht University Medical Centre (MUMC), Maastricht, Netherlands; ^5^Department of Psychosis Studies, King’s College London, King’s Health Partners, London, United Kingdom; ^6^Department of Psychiatry, UMC Utrecht Brain Centre, Utrecht University Medical Centre, Utrecht, Netherlands; ^7^Department of Psychiatry, Federal University of Rio Grande do Sul, Porto Alegre, Brazil; ^8^Departamento de Psiquiatria, Faculdade de Medicina FMUSP, Universidade de São Paulo, São Paulo, Brazil

**Keywords:** psychotic experiences, attenuated psychotic symptoms, adolescents, schizophrenia, psychiatric epidemiology

## Abstract

**Purpose:** Psychotic experiences in childhood (such as hearing voices or being suspicious) represent an important phenotype for early intervention. However, these experiences can be defined in several ways: self-reported psychotic experiences (SRPE) rely exclusively on the child’s report, clinically validated psychotic experiences (CRPE) are based on clinical assessment, and attenuated psychotic symptoms (APS) represents a categorization to do with clinical relevance in relation to severity. Very few studies have investigated how these distinctions impact clinical and other domains. The present study aims to compare SRPE, CRPE, and APS among children and adolescents.

**Methods:** This study is part of the Brazilian High-Risk Cohort Study for Psychiatric Disorders, in which 2,241 individuals aged 6–14 years provided self-ratings of 20 psychotic experiences using the Community Assessment of Psychic Experiences (CAPE). A trained psychologist conducted an interview to validate or reject reported experiences and to rate the presence of APS and affective flattening. In parallel, parents provided information about child mental health to an independent interviewer. We tested the association of mutually exclusive categories of non-validated SRPE (nSRPE), clinically validated PE below the threshold for APS (nCRPE), and APS (nSRPE = 33%, nCRPE = 11%, APS = 6%), with parents’ information about the child’s positive attributes and levels of psychopathology and psychologist assessment of blunted affect.

**Results:** Most associations were qualitatively similar, and there was a dose–response in the strength of associations across categories, such that APS > nCRPE > nSRPE. Experiences in all three categories were associated with female sex. nSRPE were associated with overall levels of psychopathology, but to a lesser degree than nCRPE and APS. APS and nCRPE were associated with less positive attributes, with APS more so than nCRPE. Only APS was associated with affective flattening.

**Conclusions:** In children and adolescents, SRPE, CRPE, and APS all index liability for psychopathology, but as clinician rated relevance increases, associations get stronger and become evident across more domains.

## Introduction

Children and adolescents who present with unusual experiences, such as perceptual a bnormalities and cognitive distortions that resemble delusions and hallucinations but are below the threshold for clinical psychosis (1, 2), are at risk of mental health problems in later life (1, 3, 4). Subclinical psychotic experiences (PE) are associated with psychiatric morbidity (5–7) and may impact global functioning to a larger extent than other mental health problems (8). In particular, PE index risk of the emergence of psychotic disorders (9), parasuicidal behaviors (10), and other mental health problems like anxiety and depression (11–13).

Because unusual experiences have been associated with risk of mental health problems, the parameter “presence of unusual experiences” has been used for the selection of individuals who may be targeted for preventive interventions. Consequently, clinical trials testing interventions to promote mental health among those who present with unusual experiences are emerging (3, 4), and at least one clinical guideline already recommends that children and adolescents with experiences of transient or attenuated psychotic symptoms should be referred to a specialist mental health service or an early intervention in psychosis service (2).

Although the evidence linking unusual experiences and negative mental health outcomes is consistent and justifies a focus on individuals at risk (8), markedly different methodologies for the assessment of such experiences exist, and there is very little empirical data comparing the different methods of assessment (14–17). Across the literature on unusual experiences, studies have used a range of different definitions: “psychotic experiences,” “self-reported psychotic experiences,” “false-positive psychotic experiences,” “clinically validated psychotic experiences,” and “attenuated psychotic symptoms” (APS). Thus, PE can be assessed by self-report and/or clinical judgment, in an attempt to identify the varying degrees to which one may present with perceptions and thoughts that resemble psychosis, yielding clinically relevant PE (CRPE) and clinically non-confirmed self-reported PE (nSRPE) (18). CRPE, however, may not fulfill criterion for APS as the identification of APS requires a more complex assessment on which a trained evaluator conducts an in-depth clinical interview and, based on that, has to judge whether subclinical delusions, hallucinations, or disorganization of speech surpasses a given level that is close to but below the threshold for clinical psychosis (19). Thus, a part of those considered to have CRPE are not classified as APS (hereafter: non-APS CRPE, or simply nCRPE). Although all measures (SRPE, CRPE, and APS) describe phenotypes that pertain to the same spectrum phenotype that also includes clinical psychosis, they came from different theoretical frameworks and have been tested across a range of different settings. PE are often assessed using self-reports in the context of epidemiological surveys and show relatively high prevalence rates in the general population, particularly in children and adolescents (20). APS, on the other hand, are assessed in a clinical context, with a particular focus on help-seeking late adolescents or young adults approaching the age for the peak incidence of psychosis (21). While PE are assumed to be present to varying degrees in non-clinical populations (22), APS are typically assessed in the context of help-seeking for disorders of anxiety, depression, and drug misuse and may even index imminent conversion to psychosis (19). In other words, although both PE and APS indicate risk, PE are very common among young people (20) and can be considered epidemiologically normative to a certain degree (23). APS, on the other hand, are thought to arise in the context of early pathology and may be suitable for the identification of individuals in need of preventive interventions (21, 24). PE severity and persistence over time may represent important moderators; prospective studies showed that higher loads of SRPE and more persistent SRPE are more strongly associated with psychopathology (18). Clinical validation of SRPE was examined in a number of studies, usually to examine the predictive validity of self-report against clinical judgment (14, 16, 17, 25).

Although PE and APS are frequently studied in the literature of risk of psychosis, there is not much work examining to what degree they reflect the same underlying dimension (21, 26, 27). Children and adolescents are a particularly relevant age group for at-risk interventions. Examination of the conceptual overlap of the different measurements in children and adolescents is thus important, considering that unusual experiences may be used to enrol individuals in clinical treatment (2) and that, depending on the measure, such phenomena are not rare among youth (28). When self-report is used, 17% of children aged 9–12 years and 7.5% of adolescents aged 13–18 years report hearing voices (20), while almost all of the children endorse at least one psychotic experience when 20 different PE are investigated (29). There is a growing literature on the validity of self-report against clinical judgment for the evaluation of PE, showing they form part of the same continuum (14, 25). However, no study has tested the results of the two diagnostic procedures in a population-based sample not limited to individuals seeking help from services (15–17).

The present study explores the rates and clinical correlates of SRPE, CRPE and APS in an epidemiological, school-based sample of children and adolescents. For this purpose, 2,241 children aged 6–14 years were assessed for self-report and clinical ratings of 20 PE. Children were further classified in terms of fulfilling or not the criteria for APS, based on applying an APS criterion from the CAARMS instrument (30). Parents were interviewed independently on child demographic characteristics, mental health, and behavior. The aim was to compare the convergent and divergent validity of SRPE, CRPE, and APS. For this purpose, we tested the strength of the association between mutually exclusive categories of non-validated SRPE (nSRPE), clinically relevant PE below the threshold for APS (nCRPE), and APS, with overall levels of psychopathology, positive attributes, and blunted affect. Hypotheses were that nSRPE, nCRPE, and APS assess different levels of severity but are pertaining to the same underlying construct, which ranges from poor mental health to more severe psychopathology, including psychosis. More specifically, based on previous work in this area (14, 18, 25, 31, 32) we expected nSRPE to be associated with levels of psychopathology (positive association) and indicators of positive attributes (negative association), but at lower levels than nCRPE. In addition, we expected APS, to a greater degree than nCRPE, to be associated additionally with psychopathology such as blunted affect, based on the observation of psychosis as a multidimensional clinical psychotic syndrome (33).

## Methods

### Study Design and Subjects

This study is part of the baseline assessment of the Brazilian High-Risk Cohort Study for Psychiatric Disorders, a cohort study of 2,511 children and adolescents in two major cities of Brazil (São Paulo and Porto Alegre). The main aim of the study was to identify developmental trajectories that lead to psychopathology, which is why children and adolescents with increased levels of familial psychopathology were oversampled. Details about the study protocol were published in a previous methodological paper (34). The project was approved by the standing ethics committee of the University of São Paulo. All parents provided written informed consent and children provided written informed assent.

In the year 2009, biological parents of all children aged 6–12 years, registered in a convenience sample of 54 public schools, were invited to participate. Parents of 9,937 children consented to participate; these children and their first-degree relatives were screened for the presence of mental disorders using the Family History Screening (FHS) (35). FHS is a structured interview conducted by lay interviewers in which parents are asked to provide information about the presence of DSM-IV major mental symptoms in each of the biological first-degree relatives. Of the 9,937 children, 1,500 were randomly selected and invited to participate and 957 responded (hereafter: “random subsample”). Children not included in the random subsample who had screened positive for mental health problems of interest were ranked according to percentage of first-degree relatives screening positive for mental disorders. Children ranked highest were recruited first and enrolment continued until the predefined maximum of 2,511 subjects was reached. This resulted in a subsample with 1,554 children with high rates of personal and familial liability for ADHD, anxiety disorders, OCD, psychotic experiences, and learning difficulties.

The 2,511 children included were further evaluated in the subsequent year, in which lay interviewers conducted household interviews with parents, while psychologists visited schools or houses to evaluate children.

### Assessments of PE and APS

The assessment of PE included the following procedures:

1. *Self-report*: Self-report was assessed using the Community Assessment of Psychic Experiences (CAPE) (36, 37). Because low levels of literacy were expected in our sample, trained psychologists read the questions of the CAPE verbatim to the children. In case children could not understand the questions, psychologists were instructed to repeat the questions up to three times exactly as displayed in the verbatim. If after three repetitions the child could not understand the question, psychologists were allowed to read the verbatim substituting some words by synonyms, but not to change the structure of the sentence or to give children explanations or examples. CAPE investigates 20 psychotic experiences, including perceptual abnormalities, persecutory ideation, bizarre experiences, and magical thinking (**Table 2** lists PE covered by CAPE). Each of the 20 items is composed of one opening question (“yes” or “no”) and two subsequent questions, on which those who endorse the symptom in the opening question are queried about the frequency and distress caused by the experience. Coding is as follows: for frequency, 1 = *“never,”* 2 = *“sometimes,”* 3 = *“frequently,”* 4 = *“nearly always”*; for distress, 1 = *“not distressing,”* 2 = *“a bit distressing,”* 3 = *“quite distressing,”* 4 = *“very distressing.”* When the opening question is coded “no,” the frequency and distress questions are coded “0.” To assert the presence or absence of PE, items were dichotomized as 0 = *“no/never/sometimes”* and 1 = *“frequently/nearly always”* for frequency items, and distress item dichotomization was as follows: 0 = *“not/a bit distressing”* and 1 = *“quite/very distressing.”*
2. *Clinical confirmation of PE*: A total of 42 psychology graduates worked on this project. All psychologists were trained by clinical psychiatrists with expertise in psychosis and in the evaluation of children and adolescents. After rating the CAPE self-report of the item read verbatim, psychologists were instructed to explore the clinical significance of the reported PE, asking details and examples about each item that screened positive. They were allowed to make a free clinical interview. Psychologists also gathered information about the child’s cultural background, the explanation the child gave for the experience, compatibility with age-related fantasies, and level of conviction associated with the experience. Based on this information, psychologists made a clinical judgment differentiating clinically relevant from clinically non-relevant experiences. To set the criterion of what was clinically relevant, psychologists were instructed to judge whether or not the reported experience would raise concern about the child’s mental health status in a clinical setting. Experiences that could be considered developmentally appropriate or that could be explained by contextual factors (e.g., imagination, fantasy, sleep-related phenomena, normal reaction of fear) should be disregarded (see **Supplementary Material** for a list of examples of experiences disregarded by clinicians for some items of CAPE). Possible scores for clinical judgment were as follows: 1 = *“unlikely to be a PE,”* 2 = “*a little likely,”* 3 = *“very likely,”* 4 = *“certainly a PE.”* When psychologists were in doubt about which score to choose, a written report about the experience reported by the child was presented for further review by a child psychiatrist. A total of 1,216 experiences were thus transcribed and reviewed. For the categorization of children in terms of having or not having CRPE, experiences considered by psychologists *unlikely* or *a little likely* were coded 0 and experiences that were *very likely* or *certainly a PE* were coded 1.
3. *Attenuated psychotic symptoms*: *APS* were assessed using the clinical rating of perceptual abnormalities, unusual mental content, and speech organization from the Comprehensive Assessment of At-Risk Mental States (CAARMS) (30). The same clinical psychologists who evaluated PE also used ratings from CAARMS to assess APS. Although CAARMS ratings were used, psychologists did not conduct the complete interview from CAARMS. Instead, after conducting a free clinical interview to inspect CAPE symptoms, they would use additional questions from CAARMS to assess symptoms not covered by CAPE, as required. Psychiatrists who had been fully trained in the instrument, and had experience in its use, trained psychologists to use CAARMS ratings. The training session included instructional information and coding exercises. CAARMS subscales have anchored scores that are as follows: 0 = “Absent,” 1 = “Questionable,” 2 = “Mild,” 3 = “Moderate,” 4 = “Moderately severe,” 5 = “Severe,” 6 = “Severe and psychotic.” Delusions can be rated in the CAARMS on one of two subscales, that is “unusual mental content” and “non-bizarre ideation” subscales. Because the anchors for both subscales are the same and individuals that are positive in one or the other will be considered positive for APS, psychologists assessed delusions in a single rating, taking into consideration all delusions displayed by the individual. As stated in the criterion for the diagnosis of “ultra-high risk” proposed by CAARMS, APS were considered sub-threshold for psychosis with a score of 3–5 on the *Unusual Thought Content/Non-bizarre ideas* subscale, a score of 3–4 on the *Perceptual Abnormalities* subscale, or a score of 4–5 on the *Disorganised Speech* subscale. Although scores higher than these are considered overt psychotic symptoms in CAARMS, the 15 individuals whose symptoms were rated as overt psychotic symptoms were nevertheless included in the APS sub-threshold group so as to not invalidate the comparison with CAPE ratings, which do not exclude the upper level of severity of psychotic experiences.
4. *Mutually exclusive categories of PE and APS*: Individuals were categorized into four mutually exclusive groups: 1) those without any PE or APS, the reference category; 2) those with at least one PE according to self-report, but no PE according to clinical evaluation nor any APS (nSRPE); 3) those with at least one PE according to clinical evaluation, but not APS (nCRPE); and 4) fulfilling criterion for APS (APS).

### Assessment of Predictors

1. *Socioeconomic status*: Socio economic status (SES) was obtained from household assets and education of the household head according to the Brazil Criterion for Economic Classification proposed by ANEP (38). Based on this criterion, families were scored between 0 and 46, resulting in classes A (35–46, the wealthiest) to E (0–7, the poorest). The C class is the middle class in Brazil, and in 2009, it corresponded to a per-capita mean monthly income of US$475 (39).
2. *Overall levels of psychopathology*: Parental ratings on two screening instruments were used to access children’s overall levels of psychopathology: the Strengths and Difficulties Questionnaire (SDQ) (40) and the Child Behaviour Checklist (CBCL) (41). Because reading proficiency is low in the Brazilian population, lay interviewers read questions for parents and took notes of their answers. The SDQ covers hyperactivity and social, emotional, and conduct problems; it allows two general scores, the “total difficulties score” and the “impact score.” The “total difficulties score” ranges from 0 to 40, higher scores representing higher levels of symptoms. The “impact score” ranges from 0 to 10, higher scores denoting higher impact of behavioral problems on daily functioning. The CBCL has 118 items that are summarized in a score that ranges from 0 to 2. It covers eight domains of psychopathology: anxious/depressed, withdrawn/depressed, somatic complaints, social problems, thought problems, attention problems, rule-breaking behavior, and aggressive behavior.
3. *DSM IV diagnosis*: The Development and Well Being Assessment (DAWBA) (42, 43) was used to assess children’s DSM-IV diagnoses of mental disorders. The DAWBA is a package of interviews, questionnaires, and rating techniques designed to generate ICD-10 and DSM-IV or DSM-5 psychiatric diagnoses on 2- to 17-year-olds. Although, ideally, DAWBA combines multiple sources of information, the report of youth is only possible for those who are 11 years or older as younger children tend to provide non-reliable reports (44). The DAWBA interviews can be administered either by interviewers or by computer, and interviewers only have to be trained in how to apply the interview (42, 43). In our study, lay interviewers interviewed only parents, and the diagnostic ratings were revised by a psychiatrist combining information from coded items and open questions.
4. *Positive attributes*: This measurement was obtained from parents’ answers on the Children Youth Strengths Inventory (YSI) from DAWBA (45), a 24-item inventory accessing positive behaviors. Examples of good behaviors covered by YSI are: being good with friends, helpful at home, and polite. Scores range from 0 to 48, higher scores indicative of more positive attributes.
5. *Estimated intelligence quotient (IQ)*: IQ was estimated using the vocabulary and block design subtests of the Weschler Intelligence Scale for Children, 3rd edition—WISC-III (46), using the Tellegen and Briggs method (47) and Brazilian norms (48).

### Statistical Analysis

Stata/SE 13.1 was used for all analyses (49). The data have a cross-level structure because assessments are clustered within both psychologists and school, the same pool of psychologists visiting all schools. Data were also clustered by site (São Paulo vs. Porto Alegre city), but because there were only two cities, a dummy for city was added to the models instead of adding an extra level to the multilevel analysis (50). Before analyzing any results, intraclass correlation coefficients of possible levels for analysis were obtained for different model structures and models were compared.

The strength of the association between different outcomes and predictors was estimated using multilevel or logistic regression models. Stata xtmixed and xtmelogit commands were used for the multilevel (cross-level) linear and logistic regression models. For all models, the dependent variables (e.g., SRPE, CRPE, and APS) and the independent variables (age, sex, SES, levels of psychopathology, positive attributes, etc.) were reversed. This was done because the previously described multilevel models are the correct method to analyze the present data, and the analysis of a categorical dependent variable in a multilevel setting is not possible in Stata. In order to avoid demographic characteristics and IQ confounding the associations, models were adjusted for age, sex, SES, and IQ.

Before answering the research question, children included and excluded from the analysis were compared in terms of association with age, sex, SES, and overall levels of psychopathology (CBCL total scores, SDQ difficulties and impact scores, and number of DSM-IV psychiatric diagnoses) and frequencies of SRPE, CRPE, and APS were described.

Comparative associations between nSRPE, nCRPE, and APS on the one hand and demographic characteristics (age, sex, and SES) and IQ on the other were tested. The four mutually exclusive groups (no PE, nSRPE, nCRPE, and APS) were recoded into dummies using “no PE” as the reference category. Subsequently, the comparative pattern of associations of nSRPE, nCRPE, and APS was probed by assessing the association of categories nSRPE, nCRPE, and APS with mental health (CBCL total scores, SDQ difficulties and impact scores, and number of DSM-IV psychiatric diagnoses) (5, 51), a measure of “positive behavioral attributes” (YSI) (45) and a measure of affective flattening, derived from the CAARMS, rated on a five-level scale of “absent,” “questionable,” “mild,” “moderate,” and “moderately severe.”

Post-estimation Wald tests were used to test differences between regression coefficients obtained from the same regression model.

## Results

### Sample Characteristics

Of the 2,511 children included in the study, 267 children did not attend psychological evaluation and four children who attended psychological evaluation had more than four items of the CAPE missing and were excluded. The 271 (10.8%) children who were excluded did not significantly differ from those who completed the evaluation in terms of demographic and clinical characteristics (age *B* = −0.16, 95% CI = −0.95 to 0.63, *p* = 0.69; sex OR = 0.9, 95% CI = 0.65–1.23, *p* = 0.5; SES *B* = 0.24, 95% CI = −1.61 to 2.09, *p* = 0.8; SDQ impact score *B* = −0.05, 95% CI = −0.41 to 0.31, *p* = 0.79; SDQ difficult scores = −0.33, 95% CI = −1.9 to 1.23, *p* = 0.68; CBCL total scores = −0.06, 95% CI = −0.14 to 0.02, *p* = 0.15; DSM-IV diagnosis *B* = 0.03, 95% CI = −0.07 to 0.13, *p* = 0.55; DAWBA positive attributes score *B* = 0.87, 95% CI = −1.56 to 3.3, *p* = 0.48).

Participants were aged 6–14 years (mean age = 10.4, SD = 1.9; **Table 1**) and 53% were male. The great majority of children, *N* = 2,010 (91%), were middle class or above. Overall levels of psychopathology were high and 27% of children had at least one DSM IV diagnosis. Mean IQ was 101.6 (SD = 16.7), which is similar to the normative mean, defined to be 100 for the general population. In general, parents rated their children’s positive attributes high (mean = 35.4, SD = 8.56). Psychologists considered that 108 children (5%) had mild to moderately severe blunted affect.

**Table 1:** Sample characteristics.

	Mean (SD) or *N* (%)
	*N* = 2,241*
**Demographics**	
Age (years)	10.4 (1.9)
Gender (percentage of male)	1,185 (52.9%)
SES	
A/B (the wealthiest)	500 (22,6%)
C	1,510 (68.2%)
D/E (the poorest)	203 (9.2%)
**General psychopathology**	
SDQ impact score (0–10, the higher the worse)	0.9 (1.5)
SDQ difficult score (0–40, the higher the worse)	14.9 (7.9)
CBCL total (0–2, the higher the worse)	0.26 (0.24)
DAWBA DSMIV (percentage per category of number of diagnosis)	
None diagnosis	1,807 (75.3%)
One	361 (15%)
Two	166 (6.9%)
Three or more	67 (2.8%)
**Intellectual functioning**	
IQ (points, the higher the better)	101.6 (16.7)
**Positive attributes**	
DAWBA Children Youth Strengths Inventory (YSI) (0–48, the higher the better)	35.4 (8.6)
**Clinical characteristic associated with psychosis**	
Blunted affect (CAARMS, 0–4, the higher the worse)	
Absent	2,015 (84.1%)
Questionable	268 (11.2%)
Mild	65 (2.7%)
Moderate	38 (1.6%)
Moderately severe	10 (0.4%)

*Due to missing data, for some variables, the total number may differ from 2,241. IQ is available for 2,239 individuals, SES for 2,213 individuals, and positive attributes for 2,240.

### Frequencies of SRPE, CRPE, and APS

**Figure 1:**
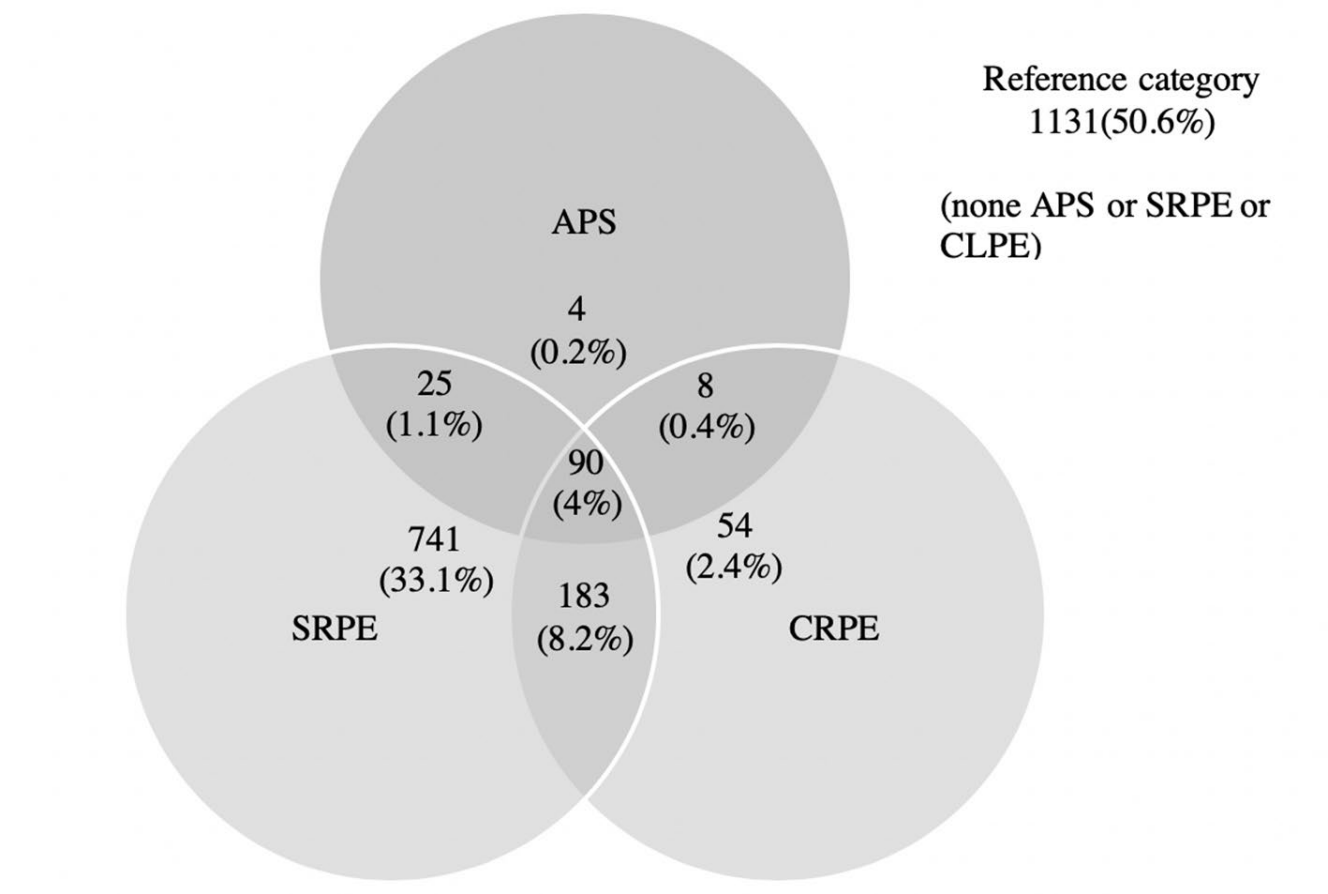
Frequencies of subjects across different categories of the presence of self-reported psychotic experiences (SRPE), clinically validated psychotic experiences (CRPE), and attenuated psychotic symptoms (APS).

The overall frequency of SRPE was high (**Table 2**). In total 1,040 (46%) children reported at least one PE that was reported at a frequency of at least “frequently” or at a distress level of at least “quite distressing.” The proportion of children with at least one PE was considerably lower if clinical judgment was used. Thus, 336 children (15%) were categorized as having at least one very likely or certain CRPE. There was a considerable overlap between categories (see **Figure 1**), and when the sample was divided into mutually exclusive categories of unusual experiences, 741 children (33%) had at least one SRPE but not CRPE or APS (here referred to as non-confirmed self-reported psychotic experiences, nSRPE), 237 children (11%) had at least one CRPE but not APS (here called non-APS clinically relevant Psychotic Experience, nCRPE), and 127 (6%) had APS (**Table 3**).

**Table 2:** Frequency of self-reported (SRPE) and clinically relevant psychotic experiences (CRPE) per item for the overall sample and subsamples according to recruitment methodology (increased familial levels of psychopathology and randomly selected).

	Overall sample		Random subsample (randomly selected)		At-risk subsample(increased intra-familial levels of psychopathology)|	
	*N* = 2,241		*N* = 864		*N* = 1,377	
CAPE item	SRPE Reported to be “frequent” or “nearly always” OR “quite” or “very distressing”	CRPE Clinically judged to be “very likely” or certainly a PE”	SRPE Reported to be “frequent” or “nearly always” OR “quite” or “very distressing”	CRPE Clinically judged to be “very likely” or certainly a PE”	SRPE Reported to be “frequent” or “nearly always” OR “quite” or “very distressing”	CRPE Clinically judged to be “very likely” or certainly a PE”
**Perceptual abnormalities**						
Do you ever hear voices when you are alone?	250 (11.2%)	129 (5.8%)	76 (8.8%)	43 (5%)	174 (12.6%)	86 (6.3%)
Do you ever hear voices talking to each other when you are alone?	75 (3.35%)	49 (2.2%)	22 (2.6%)	15 (1.7%)	53 (3.9%)	34 (2.5%)
Do you ever see objects, people, or animals that other people cannot see?	124 (5.5%)	74 (3.3%)	33 (3.8%)	19 (2.2%)	91 (6.6%)	55 (4%)
**Persecutory ideation**						
Do you ever feel as if you are being persecuted in some way?	216 (9.6%)	110 (4.9%)	73 (8.5%)	39(4.5%)	143 (10.4%)	71(5.2%)
Do you ever feel as if people seem to drop hints about you or say things with a double meaning?	91 (4%)	17 (0.8%)	36 (4.2%)	5(0.6%)	55 (4%)	12 (0.9%)
Do you ever feel as if there is a conspiracy against you?	163 (7.3%)	21 (0.9%)	52 (6%)	5 (0.6%)	111 (8%)	16 (1.2%)
Do you ever feel that people look at you oddly because of your appearance?	163 (7.3%)	19 (0.8%)	58 (6.7%)	7(0.8%)	105 (7.6%)	12 (0.9%)
Do you ever feel as if some people are not what they seem to be?	335 (15%)	14 (0.6%)	126 (14.6%)	4(0.5%)	209 (15.2%)	10 (0.7%)
Do you ever feel as if things in magazines or on TV were written especially for you?	47 (2%)	16 (0.7%)	16 (1.9%)	4(0.5%)	31 (2.3%)	12 (0.9%)
Do you ever feel as if a double has taken the place of a family member, friend or acquaintance?	14 (0.6%)	17 (0.8%)	3 (0.4%)	3 (0.4%)	11 (0.8%)	14 (1%)
**Magical thinking**						
Do you believe in the power of witchcraft, macumba, or supernatural things?	248 (11%)	14 (0.6%)	83 (9.6%)	7(0.8%)	165 (12%)	7 (0.5%)
Do you ever feel as if you are destined to be someone very important?	311 (13.9%)	10 (0.5%)	106 (12.3%)	1(0.1%)	205 (14.9%)	9 (0.7%)
Do you ever feel that you are a very special or unusual person?	192 (8.6%)	10 (0.5%)	69 (8%)	4(0.5%)	123 (8.9%)	6 (0.4%)
Do you ever think that people can communicate telepathically?	59 (2.6%)	14 (0.6%)	20 (2.3%)	3(0.4%)	39 (2.8%)	11 (0.8%)
**Bizarre experiences**						
Do you ever feel as if electrical devices such as computers can influence the way you think?	75 (3.4%)	10 (0.5%)	30 (3.5%)	5 (0.6%)	45 (3.3%)	5 (0.4%)
Do you ever feel as if the thoughts in your head are being taken away from you?	34 (1.5%)	28 (1.3%)	16 (1.9%)	12 (1.4%)	18 (1.3%)	16 (1.2%)
Do you ever feel as if the thoughts in your head are not your own?	40 (1.8%)	24 (1.1%)	18 (2.1%)	7 (0.8%)	22 (1.6%)	17 (1.2%)
Have your thoughts ever been so vivid that you were worried other people would hear them?	72 (3.2%)	42 (1.9%)	22(2.6%)	7(0.8%)	50 (3.6%)	35 (2.5%)
Do you ever had the sensation that you could hear your own thoughts?	181 (8.1%)	36 (1.6%)	71(8.2%)	9(1%)	110 (8%)	27 (2%)
Do you ever feel as if you are under the control of some force or power other than yourself?	46 (2.1%)	35 (1.6%)	16(1.9%)	8 (0.9%)	30 (2.2%)	27 (2%)
**At least one**	**1,040 (46.4%)**	**336 (15%)**	**380 (44%)**	**112 (13%)**	**660 (47.9%)**	**224 (16.3%)**

**Table 3:** Association of mutually exclusive categories of non-confirmed self-reported psychotic experiences (nSRPE), non-APS clinically relevant PE (nCRPE), and attenuated psychotic symptoms (APS) and demographic and clinical variables.

	nSRPE SRPE but not CRPE or APS	nCRPE CRPE but not APS	APS	
*N* = 2.236	741 (33%)	237 (11%)	127 (6%)	
Demographic and clinical characteristics	Regression coefficients (*B*) or odds ratio (OR) and respective confidences intervals (CI), *p* values†Tested against the reference category without SRPE or CRPE or APS (*N* = 1,131 (50.6%))			Between-coefficient differences
**Demographic variables**				
Age	*B*(1) = −0.04 (−0.23 to 0.15), *p* = 0.68	*B*(2) = −0.12 (−0.39 to 0.15), *p* = 0.38	*B*(3) = −0.16 (−0.53 to 0.21), *p* = 0.4	*B*(1) ≠ *B*(2), χ^2^ = 0.33, *p* = 0.57*B*(1) ≠ *B*(3), χ^2^ = 0.4, *p* = 0.53*B*(2) ≠ *B*(3), χ^2^ = 0.03, *p* = 0.86
Gender (0 = female, 1 = male)	OR(1) = 0.71 (0.58–0.87), *p* = 0.001***	OR(2) = 0.77 (0.57–1.03), *p* = 0.08	OR(3) = 0.63 (0.42–0.93), *p* = 0.02*	OR(1) ≠ OR(2), χ^2^ = 0.25, *p* = 0.62OR(1) ≠ OR(3), χ^2^ = 0.39, *p* = 0.53OR(2) ≠ OR(3), χ^2^ = 0.78, *p* = 0.38
SES (0–46, the higher the wealthier)	B(1) = −0.4 (−0.9 to 0.1), *p* = 0.12	*B*(2) = −0.45 (−1.15 to 0.26), *p* = 0.21	*B*(3) = −0.58 (−1.54 to 0.37), *p* = 0.23	*B*(1) ≠ *B*(2), χ^2^ = 0.01, *p* = 0.9*B*(1) ≠ *B*(3), χ^2^ = 0.14, *p* = 0.71*B*(2) ≠ *B*(3), χ^2^ = 0.06, *p* = 0.8
**Intellectual functioning**				
IQ (100 ± SD, the higher the better)	*B*(1) = −0.18 (−1.85 to 1.48), *p* = 0.83	*B*(2) = 0.62 (−1.69 to 2.94), *p* = 0.6	*B*(3) = −0.71 (−3.87 to 2.45), *p* = 0.66	*B*(1) ≠ *B*(2), χ^2^ = 0.44, *p* = 0.51*B*(1) ≠ *B*(3), χ^2^ = 0.11, *p* = 0.74*B*(2) ≠ *B*(3), χ^2^ = 0.54, *p* = 0.46
**General psychopathology (parents’ report)**				
SDQ difficult score (0–40, the higher the worse)	*B*(1) = 0.93 (0.17–1.68), *p* = 0.02*	*B*(2) = 2.41(1.3–3.5), *p* ≤ 0.001***	*B*(3) = 2.3 (0.83–3.78), *p* = 0.002**	*B*(1) ≠ *B*(2), χ^2^ = 6.33, *p* = 0.02*B(1) ≠ *B*(3), χ^2^ = 3.25, *p* = 0.07*B*(2) ≠ *B*(3), χ^2^ = 0.01, *p* = 0.91
CBCL total scores (0–2, the higher the worse)	*B*(1) = 0.03 (0–0.05), *p* = 0.04*	*B*(2) = 0.05 (0.01–0.08), *p* ≤ 0.005**	*B*(3) = 0.09 (0.05–0.14), *p* = ≤0.001***	*B*(1) ≠ *B*(2), χ^2^ = 1.72, *p* = 0.19*B*(1) ≠ *B*(3), χ^2^ = 8.09, *p* ≤ 0.005**B(2) ≠ *B*(3), χ^2^ = 2.66, *p* = 0.1
SDQ impact score (0–10, the higher the worse)	*B*(1) = 0.04 (−0.12 to 0.18), *p* = 0.65	*B*(2) = 0.31 (0.09–0.53), *p* ≤ 0.006***	*B*(3) = 0.59 (0.29–0.88), *p* ≤ 0.001***	*B*(1) ≠ *B*(2), χ^2^ = 5.42, *p* = 0.02*B(1) ≠ *B*(3), χ^2^ = 13.15, *p* ≤ 0.001***B(2) ≠ *B*(3), χ^2^ = 2.63, *p* = 0.11
DSMIV diagnosis (number of diagnosed disorders)	*B*(1) = 0.05 (−0.02 to 0.13), *p* = 0.18	*B*(2) = 0.12 (0.01–0.24), *p* = 0.03*	*B*(3) = 0.19 (0.05–0.34), *p* = 0.01**	*B*(1) ≠ *B*(2), χ^2^ = 1.41, *p* = 0.24*B*(1) ≠ *B*(3), χ^2^ = 3.4, *p* = 0.07*B*(2) ≠ *B*(3), χ^2^ = 0.64, *p* = 0.42
**Positive attributes (parents’ report)**				
DAWBA Children Youth Strengths Inventory (YSI) (0–48, the higher the better)	*B*(1) = −0.53 (−1.41 to 0.35), *p* = 0.24	*B*(2) = −1.31 (−2.55 to −0.06), *p* = 0.04*	*B*(3) = −3.48 (−5.16 to −1.8), *p* ≤ 0.001***	*B*(1) ≠ *B*(2), χ^2^ = 1.42, *p* = 0.23*B*(1) ≠ *B*(3), χ^2^ = 11.75, *p* ≤ 0.001***B(2) ≠ *B*(3), χ^2^ = 4.94, *p* = 0.03*
**Clinical characteristic associated with psychosis**				
Blunted affect (CAARMS, 0-6, the higher the worse)	*B*(1) = −0.01 (−0.07 to 0.05), *p* = 0.67	*B*(2) = 0.05 (−0.04 to 0.13), *p* = 0.29	*B*(3) = 0.22 (0.1–0.33), *p* ≤ 0.001***	*B*(1) ≠ *B*(2), χ^2^ = 1.75, *p* = 0.19*B*(1) ≠ *B*(3), χ^2^ = 15.65, *p* ≤ 0.001***B(2) ≠ *B*(3), χ^2^ = 6.8, *p* = 0.01**

^†^Modelled according to sample structure: multilevel regression models, with clinicians and schools as levels and state as a confounder. Models were adjusted for age, gender, SES, and IQ *p ≤ 0.05; **p ≤ 0.01; ***p ≤ 0.001 PE and CAARMS were evaluated by psychologists and IQ was tested by psychologists using WISC. All other measures rely on parents’ report.

### Differences Between nSRPE, nCRPE, and APS

**Table 3** displays the regression coefficients and odds ratios for the associations between the three mutually exclusive categories of nSRPE, nCRPE, and APS on the one hand and clinical characteristics on the other. When the sample was stratified by mutually exclusive category of PE status, the nSRPE group had higher SDQ difficulties and CBCL total scores than the reference group (**Table 3**). Children in the nCRPE group had higher levels of psychopathology (SDQ difficulties, impact, CBCL, and DSM-IV diagnosis) and had significantly lower ratings of positive attributes than the reference category. APS, on the other hand, were significantly associated with all the hypothesized predictors; that is, they exhibited higher levels of psychopathology, less positive attributes, and higher levels of affective flattening.

Categories were generally directionally similarly associated in a dose–response fashion, with clinical attributes in the order of APS > nCRPE > nSRPE. nCRPE was significantly more strongly associated with SDQ impact scores and SDQ difficult scores than nSRPE (**Table 3**). APS were more strongly associated with SDQ impact and difficult scores, positive attributes, and affective flattening than nSRPE. APS were associated more strongly with less positive attributes and more severe blunted affect than nCRPE.

## Discussion

The present study assessed the rates and comparative associations of SRPE, CRPE, and APS in a large non-clinical population of children at increased risk of mental disorders. The results showed that the use of clinical judgment instead of self-report resulted in an enhanced pattern of associations, but that SRPE rejected by clinicians are also associated with increased levels of general psychopathology. Also, we found that clinical validation of PE (CRPE) and higher levels of severity of PE (PE meeting APS criterion) impacted the pattern of associations with psychopathology, protective factors, and psychotic disorder-specific psychopathology. Thus, children and adolescents with CRPE had higher levels of psychopathology than those with SRPE. However, those with SRPE not validated by clinicians had higher SDQ impact scores and CBCL total scores than participants without SRPE. Another finding was that CRPE below and above the threshold for APS were not significantly different from each other in terms of associations with mental health problems in general. Although the use of the APS criterion applied to PE did not improve the prediction of psychopathology in general, its use led to the selection of a group of children with more severe affective flattening and less positive attributes. As APS, in fact, represents a severity subgroup of PE, the results support the idea of a spectrum phenotype including SRPE, CRPE, and APS, SRPE showing the weakest link to poor mental health and CRPE and APS showing stronger links with general psychopathology; however, only APS is associated with blunted affect and is more strongly linked with less positive attributes than the other two measurements.

### Self-Report Versus Clinical Evaluation of PE

Usually, clinical evaluation is considered the gold standard for the assessment of psychiatric symptoms, and this is particularly true for psychotic patients and for children. In both populations, self-report is considered unreliable because of reduced capacity of judgment. Contradicting this idea, results from the present study showed that experiences considered by children as frequent and distressing, even when judged little likely to be a PE by psychologists, were associated with increased scores of general psychopathology. In practical terms, it means that those who report frequent and distressing experiences not validated by clinicians can be, although to a lesser degree, at increased risk of mental health problems.

When self-report is used to access PE, different items tend to covary (51–53) and subdomains are highly correlated (52, 54), suggesting that all reflect the same underlying construct. This latent construct has been shown to have validity in relation to clinical psychosis; self-reported PE is associated with measures of general psychopathology, environmental risk factors for psychosis, and family history for psychotic disorders (5, 55). Although the aforementioned data supports the reliability and validity of self-reported PE, very few studies assessed the validity of self-report against clinical judgment in children and adolescents. The two available studies that investigated validity and reliability of SRPE versus CRPE found surprisingly good psychometric properties for some items of self-report questionnaires (15, 16). In these studies, the positive predictive value of the self-report of hearing voices against interview-validated auditory hallucinations was around 70% (15, 16), and its sensitivity and specificity were respectively 67% and 92% (16). Evidence from studies in adult populations have shown that disregarding PE based on clinical impression can be misleading. Adults with SRPE not validated by psychological interview have more psychiatric morbidity, substance misuse, higher levels of neuroticism, and worse affective regulation and social functioning than individuals without PE (25). Additionally, SRPE considered non-relevant by clinicians was found to prospectively predict the development of clinical psychotic disorders among adults (14). Thus, clinical evaluation might not necessarily represent the gold standard for the assessment of PE. Results from the present study showed that children that report PE considered irrelevant by clinicians might be at increased risk of mental health problems. Although this association was small and the use of clinical evaluation did improve the sensitivity of PE for the identification of children with worse metal health, the use of clinical judgment led to the exclusion of children with increased levels of psychopathology.

### Attenuated Psychotic Symptoms Versus Clinically Validated PE

Apart from the question on whether or not clinical evaluation is preferred over self-report, an additional issue on the evaluation of subclinical psychotic features is on how to set the threshold above which a given experience would be considered clinically relevant. In this study, two different strategies were tested, one broader (nCRPE) and another more exclusive (APS). From the clinical perspective, the identification of APS and CRPE follows different procedures. APS is based on setting a threshold to differentiate more severe forms of PE, and it takes into consideration not only subclinical delusions and hallucinations but also levels of speech organization. Although the use of a threshold to differentiate mild from more severe forms of PE, as is done for APS, increases the specificity of the criterion for the selection of those at higher risk of mental disorders, the threshold set by CAARMS (the most commonly used criterion for UHR) was arbitrarily defined (56). Although different authors have already proposed the use of broader phenotypes for the identification of high-risk individuals (57), to our knowledge, this study was the first to compare APS with milder forms of clinically validated PE in an epidemiological sample using clinical interviewing. We showed that, when compared to CRPE, the APS criterion failed to differentiate children with more or less severe psychopathology. Nevertheless, it was useful for the identification of a group of children with less positive attributes and more severe affective flattening. It is possible that the stronger association with affective flattening found for APS when compared to CRPE, which showed associations in the same direction but not statistically significant, is due to the fact that, differently from CRPE, APS considers information about severity or about speech organization, another characteristic of psychosis. This result is consistent with the model previously proposed, according to which PE represent transdiagnostic features and the combination of symptoms from different diagnostic constructs (i.e., positive symptoms, negative symptoms, and disorganization) allow for a more accurate classification of individuals into the categorical diagnoses of psychosis (58). Under this framework, the use of UHR criteria, combining not only PE but also other features of psychosis, can be considered an advantage in decreasing the pluripotency of the diagnosis towards a more specific phenotype to target prediction for psychosis (59).

### Association of PE With Psychopathology and Positive Attributes

Previous studies showed an association between PE and general levels of psychopathology (13, 55, 60, 61). In accordance with this earlier work, we found that the validity of PE as an indicator of mental health problems is independent of the source of assessment of PE, after correction for IQ and demographic characteristics. The association with psychopathology in our sample does not seem to be specific, with both internalizing and externalizing mental health problems being associated with the three categories (see **Supplementary Material** for frequencies of DSMIV diagnosis by mutually exclusive categories of non-confirmed self-reported psychotic experiences (nSRPE), non-APS clinically relevant PE (nCRPE), and attenuated positive symptoms (APS) and for the association of CBCL subscales with these groups). We also showed that children and adolescents with increased rates of PE not only have increased levels of psychopathology but also display fewer positive attributes and that levels of positive attributes are important in the distinction between those with more and less severe subclinical psychotic features. This is of relevance for clinical practice because the psychiatric evaluation of children frequently emphasizes more the identification of symptoms and deficits rather than on the identification of abilities, even though positive attributes may act independently from psychopathology in promoting adaptation and functioning (62). Therefore, levels of positive attributes should be further studied as a moderator of risk of mental disorders.

### Assessment of Self-Reported PE Among Young Children

There is evidence from previous research showing that, when tested against clinical judgment, young children are not reliable for the report of a range of psychiatric symptoms (44, 63). Although there is no available data on the validity of CAPE among young children, CAPE is frequently used to assess PE in youth, showing very good psychometric properties (64, 65). Indeed, CAPE seems to be more reliable among younger people. A meta-analysis of CAPE psychometric properties identified 111 studies and showed that the mean alpha values for studies with mean age lower than 25 years were more internally reliable than that of adults older than 25 years. In our sample, we found evidence that CAPE can be reliably assessed in younger children. First, the age of those with non-confirmed SRPE was not significantly lower than the age of those with clinically relevant PE (*B* = −0.08 (−0.33 to 0.17), *p* = 0.55; regression coefficients (*B*) and respective confidence intervals [CI], obtained from multilevel regression model testing non-confirmed SRPE against the reference category Clinically Relevant PE [*N* = 1,102] and adjusted for gender, socioeconomic status, and IQ). Secondly, the positive predictive value (PPV) of self-report against clinical judgment did not seem to increase with age (PVV was 27.5% for children bellow or equal 10 years old and 25.2% for older children—see **Supplementary Material** for more details about PPV of SRPE against clinical judgment and CRPE against APS). Although the significance of the difference between different values of PPV cannot be statistically tested, it was surprising to us that younger children had slightly better values of PPV. We do not believe, however, that younger children would be more reliable than the older ones in self-reporting PE; instead, it may reflect the better quality of interviews provided by older children. The evaluation of PE in pediatric population may be very challenging as children’s description of delusions are usually poorly elaborated and vague and may be built on real experiences (e.g., being teased or disliked), hallucinations are often multimodal, and children may give them names, which may be stereotypic (e.g., “the devil”) (66). Third, the internal consistency of CAPE self-report was surprisingly good when tested for the subsample of very young children (Cronbach’s alpha = 0.9 for children bellow 8 years, *N* = 259). It is possible that the fact that psychologists read CAPE questions to children increased the quality of children’s self-report, but there is also a possibility that young children can reliably provide information about their PE using CAPE self-ratings.

## Limitations

This study has some limitations that should be addressed. First, the majority of children studied were selected based on having increased risk of mental health problems. Consequently, the associations with morbidity found here can be stronger than expected for the general population. Second, psychologists’ judgment relied exclusively on interview with children; parents were not interviewed by the psychologists. Consequently, only PE reported by children were evaluated clinically and potential false negatives were not assessed.

Third, it is also important to comment some methodological caveats involving the measurements used. The context and the methodology used to assess APS in the current epidemiological population-based setting are different from the one typically used in UHR studies with a focus on selected help-seeking individuals in the clinical setting. Different from most frequently used instruments for UHR, the precise frequency of symptoms was not considered because children and adolescents were not expected to reliably report the frequency of their experience with such a precision as required by the instruments for UHR. If the frequency of experiences had been taken into consideration, even more CRPE would have been rejected. Another important difference is the fact that the psychologists who performed the clinical evaluation received 4 h of training on CAARMS ratings; they were not experts in the evaluation of prodromal schizophrenia, as would be the case in an UHR clinical setting. Furthermore, it should be mentioned that the diagnosis of UHR considers not only APS but also other features like genetic risk and deterioration of global functioning; however, these are rare and the great majority of UHR, in fact, is based on APS (67). Furthermore, we used CAARMS ratings, but not the entire clinical interview, as it would not be feasible in such a large population-based study. Consequently, the methodology used for the assessment of APS was not as rigorous as that used in prodromal clinic studies. Another issue is that the CAPE Portuguese version was not validated previously, and although there is data showing that the CAPE has very good psychometric properties for adult populations (65), it has not yet been validated for the assessment of PE in children as young as some of the children in our sample (64). Furthermore, we had someone reading the questions to the children, which may limit the generalizability of the findings to samples where youth are administered written queries of PE. We also did not collect data showing the interrater reliability of psychologist assessment of CAPE and CAARMS. Besides these possible methodological caveats, the three instruments showed good psychometric properties in our sample. The internal consistency of CAPE self-report items was very good, with an alpha of 0.89, and the exclusion of none of the items would lead to better results. When items were dichotomized to allow classification of subjects into those with and without SRPE, the internal consistency decreased, but was still acceptable, alpha = 0.74, with the exclusion of none of the items leading to better alpha values. The internal consistency of the CAPE clinical scores was also very good, with an alpha of 0.83. When items were dichotomized, the alpha values decreased, but were still acceptable (alpha = 0.72), and the exclusion of none of the items would result in better alpha values. The internal consistency of the two items of CAARMS used to generate the APS criterion was also good (alpha = 0.81). It is also noteworthy that all the three measurements were found to be associated with hypothesized predictors, as shown by the present research, which reinforces the validity of the measurements used.

Finally, although PE were associated with measurements of poor mental health, the evidence presented here is insufficient to support the idea that all children with PE should be assessed clinically. For this aim, it would be important to estimate the negative and positive predictive validity of PE as an index of future and present mental health problems as well as to compare it with other indicators and to raise empirical data on the cost-efficacy, effectiveness, and ethics of interventions to improve mental health in those with such features.

## Conclusions

In children and adolescents, self-reported PE not recognised by clinicians, clinically validated PE, and APS appear to tap into the same underlying construct, differing in predicted ways in degree and in kind as a function of clinical severity in terms of associations with psychopathology, positive attributes, indicators of the multidimensional psychotic syndrome, and demographics.

## Ethics Statement

The studies involving human participants were reviewed and approved by Comissão para análise de projetos de pesquisa (CAPPesq) da diretoria clínica do Hospital das Clínicas da Faculdade de Medicina da Universidade de São Paulo. Written informed consent to participate in this study was provided by the participants’ legal guardian/next of kin.

## Author Contributions

TM conceived and designed the study, conceived and designed the analysis, collected the data, performed the analysis, and wrote the paper. JvO and MD conceived and designed the analysis, performed the analysis, and wrote the paper. AG, PP, GS, GM, EM, LR, GP, and RB conceived and designed the study, collected the data, and provided critical feedback on the manuscript. JM and PM conceived and designed the study and provided critical feedback on the manuscript.

## Funding

This work is supported by the National Institute of Developmental Psychiatry for Children and Adolescents, a science and technology institute funded by Conselho Nacional de Desenvolvimento Científico e Tecnológico (CNPq; National Council for Scientific and Technological Development; grant number 573974/2008-0) and Fundação de Amparo à Pesquisa do Estado de São Paulo (FAPESP; Research Support Foundation of the State of São Paulo; grant number 2008/57896-8). It was also supported in part by the European Community’s Seventh Framework Program (grant agreement No. HEALTH-F2-2009-241909, Project EU-GEI). The author’s scholarships are supported by the following Brazilian government institutions: CNPq, FAPESP, Coordenação de Aperfeiçoamentode Pessoal de Nível Superior (CAPES; Brazilian Federal Agency for Support and Evaluation of postgraduate education) and Fundação de Amparo a Pesquisa do Estado do Rio Grande do Sul (FAPERGS; Research Support Foundation from the State of Rio Grande do Sul). Tais Moriyama received a sandwich scholarship from CAPES (process number 10553/12-6) and a PhD scholarship from CAPES; Ary Gadelha received a CAPES PhD scholarship; Pedro Mario Pan received a CNPq/CAPES master’s degree scholarship; Giovanni Abrahão Salum received a CAPES/FAPERGS post-doctoral scholarship; Gisele Gus Manfro, Jair J. Mari, EurípedesConstantino Miguel (302463/2011-9), Luis A. Rohde, Guilherme V. Polanczyk (310582/2017-2) and Rodrigo A. Bressan are in receipt of a senior research CNPq scholarship.

## Conflict of Interest

AG has been a member of the speakers’ bureau/advisory board and/or acted as a consultant for Janssen-Cilag, Daiichi-Sankyo and Aché. PP received a CNPq PhD scholarship. LR has been a member of the speakers’ bureau/advisory board and/or acted as a consultant for Eli-Lilly, Janssen-Cilag. He also received travel awards from Novartis and Shire to attend the 2016 AACAP and the 2018 APA meetings. GP is employed by the University of São Paulo. He has received grant or research support from the Conselho Nacional de Desenvolvimento Científico e Tecnológico (CNPq), the Fundação de Amparo à Pesquisa do Estado de São Paulo (FAPESP), the Fundação Maria Cecilia Souto Vidigal (FMCSV), Grand Challenges Canada, and the Bill and Melinda Gates Foundation. He has served as a consultant to Shire, Teva, and Medice. He has received royalties from Editora Manole. RB reports grants, personal fees, and non-financial support from Janssen and personal fees from Pfizer and from Ache outside the submitted work.

The remaining authors declare that the research was conducted in the absence of any commercial or financial relationships that could be construed as a potential conflict of interest.
