# Supplementary material for: Differences Between Self-Reported Psychotic Experiences, Clinically Relevant Psychotic Experiences, and Attenuated Psychotic Symptoms in the General Population
Source: Front Psychiatry. 2019 Oct 29;10:782. doi: 10.3389/fpsyt.2019.00782 (PMC6829673; doi:10.3389/fpsyt.2019.00782)
Supplement: Supplementary file 1 [file Table_1.docx]

| Supplementary table 1: Examples of situations reported by children as frequent of distressing but considered by psychologists as unlikely or not very likely to be a clinically relevant PE | |
| --- | --- |
| **CAPE item** | **Examples** |
| Do you ever hear voices when you are alone? | - The experience was disregarded if the child report hearing voices exclusively when initiating or during sleep (e.g. “Sometimes when I am falling asleep, I hear the sounds of children laughing”). - Experiences that can be better explained by developmentally appropriate fantasies (e.g. sometimes when I am playing with my dolls I have the feeling that I can talk to them, but I know this is just my imagination”). - Experiences that are coherent to the child’s faith and cultural background (e.g. the child believes in spirits and has the impression that the voice of her deceased grandfather could be heard among the many other voices singing ‘happy birthday’ on her birthday party). - Experiences that are very common in the general population (e.g. episodically the child has the impression that her name was whispered when actually there is nobody around). - Experiences that are contextually appropriate, like being over-sensitive to normal environmental sounds when afraid (e.g. “when I am left alone at home I get afraid and sometimes I have the feeling that someone knocked on my door when nobody was there). |
| Do you ever hear voices talking to each other when you are alone? | - Psychologists disregarded experiences that described a very specific and circumscribed situation (e.g. “it once happened that I had the impression that I heard my mother chatting when she actually wasn’t at home”). - Experiences that happens exclusively when the child is falling asleep or sleeping (see item above for examples). - The child seemed to misunderstand the questions and reported hearing real voices (“when someone talks on the street, from the living room I can hear them through the window”). - Experiences that are contextually appropriate, like being over-sensitive to normal environmental sounds when afraid (see above for examples). |
| Do you ever feel as if things in magazines or on TV were written especially for you? | - Experiences that can be culturally appropriate (e.g. a teenager who believes in horoscope predictions said “when I read the horoscope in teenager magazines, I feel like they were made for me” or an evangelical child said: “when I am watching the Gospel channel, sometimes I feel like it has a special message for me”). - Misinterpretations of the question (i.e.: “When I watched the movie “Home Alone” I remember the day my mother forgot me alone on the shore”, “once I watched this documentary about people who have high cholesterol levels that refers to something that is related to me, because I also have high cholesterol levels”). |
| Do you believe in the power of witchcraft, macumba or supernatural things? | - Experiences that can be culturally appropriate were disregarded, this was particularly relevant for macumba, a spiritual tradition common in Brazil that involves the belief in spirits and rituals with an aim to interact with them, asking them to favor or work against others, examples of experiences reported by children: “My father does macumba regularly”- in this case the family were cultivating this tradition, or “I saw people doing it on the street in front of my house once, those people could have at least cleaned the candles they used”) - Believes that could be better explained by superstitions (e.g. “I don’t like Fridays because it is the day of the curse of the black cat” – making reference to the superstition that bad things can happen if a black cat crosses your way on a Friday) or by legends that are common among children (“I believe in the legend of the Bloody Marry, I saw it on a TV show”), or that are shared with other people who believe in supernatural things (“I like to watch those TV shows about supernatural phenomena”) |
