# Supplementary material for: Differences Between Self-Reported Psychotic Experiences, Clinically Relevant Psychotic Experiences, and Attenuated Psychotic Symptoms in the General Population
Source: Front Psychiatry. 2019 Oct 29;10:782. doi: 10.3389/fpsyt.2019.00782 (PMC6829673; doi:10.3389/fpsyt.2019.00782)
Supplement: Supplementary file 2 [file Table_2.docx]

| Supplementary table 2. Frequencies of DSMIV diagnosis by mutually exclusive categories of non-confirmed Self-Reported Psychotic Experiences (nSRPE), non-APS Clinically Relevant PE (nCRPE) and Attenuated Positive Symptoms (APS) | | | | | | | | | | |
| --- | --- | --- | --- | --- | --- | --- | --- | --- | --- | --- |
| N=2,236 | | **Reference***  N=1,131  (without nSRPE, nCRPE and APS) | | **nSRPE**  N=741 | | | **nCRPE**  N=237 | | **APS**  N=127 | |
| **Anxiety disorders** |  | |  | |  |  | |  | |  |
| Separation anxiety | | 22 (2%) | | 27 (3.6%) | | | 9 (3.8%) | | 8 (6.3%) | |
| Specific phobia | | 47 (4.2%) | | 24 (3.2%) | | | 6 (2.5%) | | 4 (3.2%) | |
| Social phobia | | 13 (1.2%) | | 6 (0.8%) | | | 1 (0.4%) | | 2 (1.6%) | |
| Panic disorder /Agoraphobia | | 2 (0.2%) | | 3 (0.3%) | | | 0 | | 0 | |
| Generalized anxiety disorder | | 20 (1.8%) | | 19 (2.6%) | | | 3 (1.3%) | | 0 | |
| Post-traumatic stress disorder | | 9 (0.8%) | | 6 (0.8%) | | | 4 (1.7%) | | 1 (0.8%) | |
| Obsessive compulsive disorder | | 3 (0.3%) | | 2 (0.3%) | | | 0 | | 1 (0.8%) | |
| Other anxiety disorders | | 22 (2%) | | 12 (1.6%) | | | 4 (1.7%) | | 4 (3.2%) | |
| **Mood disorders** |  | |  | |  |  | |  | |  |
| Major depression | | 31 (2.7%) | | 21(2.8%) | | | 9 (3.8%) | | 2(1.6%) | |
| Other depression | | 4 (0.3%) | | 5(0.6%) | | | 55 (4%) | | 12 (0.9%) | |
| Anxiety depressive disorder | | 3 (0.3%) | | 1(0.1%) | | | 1 (0.4%) | | 1 (0.8%) | |
| Mania/ bipolar disorder | | 2 (0.2%) | | 2(0.3%) | | | 0 | | 0 | |
| **Attention deficit hyperactivity disorder (ADHD)** | |  | |  | | |  | |  | |
| ADHD combined subtype | | 37 (3%) | | 33(4.4%) | | | 13 (5.5%) | | 6 (4.7%) | |
| ADHD inattentive subtype | | 41 (3.6%) | | 29 (3.9%) | | | 12 (5.1%) | | 8 (6.3%) | |
| ADHD hyperactive impulsive subtype | | 18 (1.6%) | | 11 (1.5%) | | | 4 (1.7%) | | 3 (2.4%) | |
| ADHD unspecified | | 17 (1.5%) | | 10 (1.4%) | | | 5 (2.1%) | | 2 (1.6%) | |
| **Disruptive disorders** | |  | |  | | |  | |  | |
| Oppositional defiant disorder | | 54 (4.77%) | | 41 (5.5%) | | | 10 (4.2%) | | 14 (11%) | |
| Conduct disorder | | 14 (1.2%) | | 17 (2.3%) | | | 5 (2.1%) | | 1 (0.8 %) | |
| Other disruptive disorder | | 3 (0.3%) | | 2 (0.3%) | | | 0 | | 1 (0.8%) | |
| **Other diagnosis** | |  | |  | | |  | |  | |
| Autism spectrum disorders | | 8 (0.7%) | | 2 (0.3%) | | | 0 | | 2 (1.6%) | |
| Tic disorder | | 6 (0.5%) | | 6 (0.5%) | | | 2 (0.8%) | | 3 (2.4%) | |
| Eating disorders | | 3 (0.3%) | | 5 (0.7%) | | | 1 (0.4%) | | 1 (0.8%) | |
| Other | | 32 (2.8%) | | 24 (3.2%) | | | 9 (3.8%) | | 6 (4.7%) | |
| **At least one** | | **297 (25.4%)** | | **195 (26.3%)** | | | **70 (29.5%)** | | **42 (33.1%)** | |
| DSM IV diagnosis made using DAWBA interviews with parents, combined with information from coded items and open questions. Diagnostic ratings were revised by a psychiatrist. | | | | | | | | | | |
