# Supplementary material for: Differences Between Self-Reported Psychotic Experiences, Clinically Relevant Psychotic Experiences, and Attenuated Psychotic Symptoms in the General Population
Source: Front Psychiatry. 2019 Oct 29;10:782. doi: 10.3389/fpsyt.2019.00782 (PMC6829673; doi:10.3389/fpsyt.2019.00782)
Supplement: Supplementary file 3 [file Table_3.docx]

| Supplementary table 3. Association of mutually exclusive categories of non-confirmed self-reported Psychotic Experiences (nSRPE), clinically relevant PE below the threshold for APS (nCRPE) and Attenuated Psychotic Symptoms (APS) and CBLC subscales | | | | |
| --- | --- | --- | --- | --- |
|  | **nSRPE**  SRPE but not CRPE or APS | **nCRPE**  CRPE but not APS | **APS** |  |
| N=2,236 | 741 (33%) | 237 (11%) | 127 (6%) |  |
| **Demographic and clinical characteristics** | Regression coefficients (B) and respective confidences intervals (CI), p values  Tested against the reference category without SRPE or CRPE or APS (N= 1,131 (50.6%)) | | | Between coefficient differences |
| **CBCL subscales** |  |  |  |  |
| Internalizing | B(1)=0.01(–0,02-0.03), p=0.68 | B(2)=0.03(–0.01-0.06), p=0.19 | B(3)=0.08(0.03-0.13), p=0.002** | B(1)≠B(2) chi2=0.95, p=0.33  B(1)≠B(3) chi2=7.95 p=0.005*  B(2)≠B(3) chi2=3.33, p=0.07 |
| Externalizing | B(1)=0.02(–0.002-0.05), p=0.07 | B(2)=0.05(0.01-0.09), p=0.009** | B(3)=0.08(0.03-0.13), p=0.002** | B(1)≠B(2) chi2=1.71, p=0.19  B(1)≠B(3) chi2=4.58, p=0.03*  B(2)≠OR(3) chi2=1, p=0.32 |
| Total scores excluding thought problems | B(1)=0.02 (0-0,05), p=0.05* | B(2)=0.05 (0.01-0.09), p=0.007** | B(3)=0.09 (0.04-0.14), p<=0.001*** | B(1)≠B(2) chi2=1.53 p=0.22  B(1)≠B(3) chi2=6.21, p=0.01**  B(2)≠B(3) chi2=1.85, p=0.17 |
| Thought problems | B(1)=0.2 (-0.01-0.03), p=0.16 | B(2)=0.03 (0-0.06), p=0.04* | B(3)=0.09 (0.05-0.13), p<=0.001*** | B(1)≠B(2) chi2=1.05 p=0.31  B(1)≠B(3) chi2=12.9, p<=0.001***  B(2)≠B(3) chi2=6.1, p=0.01** |
| Modelled according to sample structure: multilevel regression models, with clinicians and schools as levels and state as a confounder, models were adjusted for age, gender SES and IQ;  * p value ≤ 0.05; ** p value ≤ 0.01; *** p value ≤ 0.001  PE were evaluated by psychologist and IQ was tested by psychologists using WISC, all other measures rely on parent’s report | | | | |
