# Supplementary material for: Differences Between Self-Reported Psychotic Experiences, Clinically Relevant Psychotic Experiences, and Attenuated Psychotic Symptoms in the General Population
Source: Front Psychiatry. 2019 Oct 29;10:782. doi: 10.3389/fpsyt.2019.00782 (PMC6829673; doi:10.3389/fpsyt.2019.00782)
Supplement: Supplementary file 4 [file Table_4.docx]

| Supplementary table 4. Positive Predictive Value (PPV) of self-report versus clinically confirmed PE, and of clinically confirmed PE versus APS for the whole sample and for age groups. | | |
| --- | --- | --- |
|  | **Self-reported PE versus Clinically confirmed PE**  **PPV** | **Clinically confirmed PE versus APS**  **PPV** |
| Children below or equal to 10 years old | 27.5% | 31.5% |
| Children above to 10 years old | 25.2% | 26.9% |
| **All** | **26.3%** | **29.3%** |
